# Microglia shield the murine brain from damage mediated by the cytokines IL-6 and IFN-α

**DOI:** 10.3389/fimmu.2022.1036799

**Published:** 2022-10-28

**Authors:** Phillip K. West, Barney Viengkhou, Iain L. Campbell, Markus J. Hofer

**Affiliations:** School of Life and Environmental Sciences, Charles Perkins Centre and the Sydney Institute for Infectious Diseases, The University of Sydney, Sydney, NSW, Australia

**Keywords:** microglia, interleukin-6 (IL-6), interferon-alpha (IFN-α), PLX5622, central nervous system, neuroinflammation, calcification, depletion

## Abstract

Sustained production of elevated levels of the cytokines interleukin (IL)-6 or interferon (IFN)-α in the central nervous system (CNS) is detrimental and directly contributes to the pathogenesis of neurological diseases such as neuromyelitis optica spectrum disorders or cerebral interferonopathies, respectively. Using transgenic mice with CNS-targeted production of IL-6 (GFAP-IL6) or IFN-α (GFAP-IFN), we have recently demonstrated that microglia are prominent target and effector cells and mount stimulus-specific responses to these cytokines. In order to further clarify the phenotype and function of these cells, we treated GFAP-IL6 and GFAP-IFN mice with the CSF1R inhibitor PLX5622 to deplete microglia. We examined their ability to recover from acute microglia depletion, as well as the impact of chronic microglia depletion on the progression of disease. Following acute depletion in the brains of GFAP-IL6 mice, microglia repopulation was enhanced, while in GFAP-IFN mice, microglia did not repopulate the brain. Furthermore, chronic CSF1R inhibition was detrimental to the brain of GFAP-IL6 and GFAP-IFN mice and gave rise to severe CNS calcification which strongly correlated with the absence of microglia. In addition, PLX5622-treated GFAP-IFN mice had markedly reduced survival. Our findings provide evidence for novel microglia functions to protect against IFN-α-mediated neurotoxicity and neuronal dysregulation, as well as restrain calcification as a result of both IL-6- and IFN-α-induced neuroinflammation. Taken together, we demonstrate that CSF1R inhibition may be an undesirable target for therapeutic treatment of neuroinflammatory diseases that are driven by elevated IL-6 and IFN-α production.

## Introduction

Microglia are the resident macrophages of the CNS and have multifaceted roles in physiological homeostasis in development and adulthood (1). However, these cells are best known for their role in mediating host defense against pathogens or cellular damage or injury (2). Accordingly, microglia are highly responsive to inflammatory signals produced during insult or injury and play a protective role (3–10). On the other hand, inflammatory signals in other contexts can cause these cells to lose their supportive functions and instead contribute to pathogenesis of neuroinflammatory and neurodegenerative diseases (11–17). Thus, microglia have helpful or harmful roles depending on the nature and setting of their stimuli. Importantly, the function and survival of microglia are critically regulated by colony-stimulating factor 1 receptor (CSF1R) signaling, which can be triggered by the binding of the endogenous brain growth factors CSF1 or (IL)-34 (18). The compound PLX5622 is a CSF1R inhibitor which rapidly ablates microglia in the CNS of wildtype (WT) mice, with 90% reduction of microglial cell numbers within five days of treatment (19). The depletion efficacy of CSF1R inhibitors strongly depends on the dosage and treatment duration (20, 21). Microglia undergo apoptosis following CSF1R inhibition yet, surprisingly, the loss of large numbers of microglia is not associated with elevated cytokines or other inflammatory factors, blood-brain barrier disruption, or negative effects on behavior or cognition in healthy adult mice (18, 20). Furthermore, this compound has minor off-target effects in the CNS, with minimal regulation of only a small number of genes associated with other CNS-resident cells (21). The microglia remain depleted as long as the animals are treated with PLX5622 (19) and following the termination of PLX5622 treatment, surviving microglia rapidly proliferate and repopulate the depleted CNS niche (22). Thus, acute and long-term therapeutic targeting of microglia by the administration of CSF1R inhibitors can be used to study the effect of microglial cell loss in an array of neuropathological conditions.

Neuromyelitis optica spectrum disorder (NMOSD) is an autoimmune disease characterized by optic neuritis and inflammatory lesions in the CNS due to the production of autoantibodies which target the astrocyte water channel aquaporin 4 (23). Individuals with this disease have increased levels of the cytokine IL-6 in the cerebrospinal fluid (CSF) (24–27), astrocyte damage and loss (28), reactive gliosis, demyelination (23, 29–31) and can exhibit ataxia and seizures (32, 33). On the other hand, cerebral interferonopathies are a group of disorders characterized by the appearance of clinical signs of chronic viral infection in the absence of an infectious agent. Aicardi-Goutières syndrome (AGS) is the prototypical cerebral interferonopathy and individuals with AGS have increased levels of the cytokine interferon (IFN)-α in the CSF (34–36), progressive encephalopathy with cerebral atrophy (35, 37), calcification in the basal ganglia and thalami (35, 37, 38), microangiopathy (38), diffuse white matter disease (35, 37, 38) and clinical signs such as irritability, ataxia, epileptic seizures and increased mortality (39). The pathogenesis of NMOSD and cerebral interferonopathies are causally linked to the respective actions of IL-6 and IFN-α (40, 41). Importantly, transgenic mice with sustained production of IL-6 or IFN-α under control of the astrocyte glial fibrillary acidic protein (GFAP) promoter, termed GFAP-IL6 and GFAP-IFN mice, recapitulate the cardinal clinical and pathological phenotypes of these cytokine-driven diseases (41–43) and serve as good models to dissect the neurobiological actions of IL-6 and IFN-α *in vivo*. Microglia are important target and effector cells of these cytokines in the CNS. We recently demonstrated in the GFAP-IL6 and GFAP-IFN mice that the response of microglia to IL-6 versus IFN-α is stimulus-specific and causes these cells to undergo unique adaptations to their surrounding milieu (44).

Since microglia respond to IL-6 and IFN-α *in vivo* by making extensive and dramatic changes to their morphology, distribution, turnover, transcriptome and molecular profile (40, 44), we questioned whether these alterations are indicative of distinct changes to their functional roles. Further, we investigated whether microglia help or harm the brain in the neuroinflammatory diseases driven by IL-6 versus IFN-α. To address this, GFAP-IL6 and GFAP-IFN mice were treated with the CSF1R inhibitor PLX5622 to deplete microglia and we examined their ability to recover from acute microglia depletion, as well as the impact of chronic microglia depletion on the progression of disease.

## Materials and methods

### Animals and ethics

All animal experiments were performed in compliance with the NSW Animal Research Act and its associated regulations and the 2013 NHMRC Australian code for the care and use of animals for scientific purposes. Ethical approval for the use of all mice was obtained from the animal ethics committee of the University of Sydney (2018/1428). The GFAP-IL6 (B6.Cg-Tg(Gfap-Il6)G167Lms/Niusy MGI:7327600) and GFAP-IFN mice (B6;C-Tg(Gfap-Ifna1)39Ilc/Niusy MGI:7328531) were described previously (42, 43, 45) and were bred and maintained under specific-pathogen-free conditions at the animal facility of the University of Sydney. Both GFAP-IL6 and GFAP-IFN mice were originally developed by I. L. Campbell at the Scripps Research Institute, La Jolla, CA, USA and breeding stock were obtained from there. GFAP-IL6 mice are on C57BL/6 background and GFAP-IFN mice are on a mixed C57BL/6 x BALB/c background. Wildtype littermates from both GFAP-IL6 and GFAP-IFN lines were used as WT controls and no differences were found between the WT haplotypes. Male and female mice were used in all experiments and no sex differences were observed. Animals received food and water *ad libitum*. The temperature and humidity parameters in animal holding areas were set to fall between 20-24°C and 40-70% respectively, with light between 0545 and 1745 hours. Mice were housed at a maximum density of six mice per cage. Transgenic GFAP-IL6 and GFAP-IFN colonies were assigned a clinical score and weighed weekly. Clinical scores were assigned according to the following scoring criteria: 0 = normal, 0.5 = minor ataxia, 1= altered gait, 1.5 = severely altered gait, 2 = ataxia, 2.5 = reduced activity, 3 = wild running or jumping, 3.5 = absent seizure, 4 = brief convulsive seizure with recovery, 5 = continuous convulsive seizure or found dead. A cumulative score of 5 met euthanasia criteria. Mice were euthanized with isoflurane and brains were collected and dissected at the midline. For histological analysis, hemibrains were fixed in neutral buffered 4% paraformaldehyde overnight at 4°C and then paraffin embedded. For RNA isolation and quantitative real-time PCR (qPCR), cerebella were dissected and flash frozen.

### CSF1R inhibition

PLX5622 was provided by Plexxikon Inc. and formulated in AIN-76A standard chow by Research Diets Inc. at a dose of 1200 mg/kg. One-month-old animals were fed control AIN-76A or PLX5622-containing chow *ad libitum*. For microglial cell repopulation experiments, mice were fed control or PLX5622 chow for 14 days and then PLX5622 was withdrawn for the specified time periods. For long-term microglial cell ablation experiments, mice were fed control or PLX5622 chow for 12 weeks.

### Histology

Paraffin sections (12 μm thick for microglia stains and 5 μm thick for all other stains) were deparaffinized and rehydrated in graded ethanol. Routine histology (hematoxylin and eosin (H&E), luxol fast blue and cresyl violet (LFB&CV) and alizarin red S (ARS) stains) was performed at the Histopathology Core Facility (Department of Pathology, University of Sydney). For immunohistochemistry, antigens were unmasked with 25 mM Tris pH 8, 5 mM EDTA pH 8 and 0.05% (w/v) SDS (Iba1, laminin, neurofilament-200 (NF-200), calbindin, parvalbumin), 25 mM Tris pH 9 (GFAP) or 10 mM citrate pH 6 in 0.05% Tween-20 (CD3) in a vegetable steamer for 40 min. Sections were incubated in 0.3% peroxidase for 10 min and blocked in 1% goat serum with 0.1% Triton X-100 and 0.05% Tween-20 in phosphate-buffered saline (PBS) for 30 min. The primary antibodies rabbit anti-Iba1 (019-19741, Wako Pure Chemical Industries, 1:500), rabbit anti-GFAP (Z0334, Agilent, 1:1000), rabbit anti-laminin (L9393, Sigma-Aldrich, 1:25), mouse anti-NF-200 (N0142, Sigma-Aldrich, 1:200), mouse anti-calbindin (c9848, Sigma-Aldrich, 1:1000), mouse anti-parvalbumin (P3088, Sigma-Aldrich, 1:1000) and rabbit anti-CD3 (ab16669, Abcam, 1:200) were incubated overnight at 4°C. Sections were washed in PBS and then incubated with biotinylated anti-rabbit or anti-mouse antibodies (BA-1000 and BA-2000, Vector Laboratories, 1:200) for 30 min at room temperature (RT), followed by VECTASTAIN Elite ABC HRP Kit (PK-7200, Vector Laboratories) for 30 min at RT. Sections were developed with 3,3’-diaminobenzidine with nickel enhancement (SK-4100, Vector Laboratories), counterstained with Mayer’s hematoxylin and mounted. Sections were viewed with a DM4000B microscope (Leica Microsystems) and imaged using a SPOT Flex 15.2 64 Mp Shifting Pixel camera and SPOT Advanced 4.5 software (Diagnostic Instruments). The number of immunolabelled cells was counted using the Cell Counter plugin in ImageJ software (NIH, USA). For the ARS stains, calcification scores were assigned according to the following scoring criteria: 0 = no calcification, 1 = small, isolated single calcified deposits, 2 = multiple calcified deposits in a single group, 3 = multiple groups of deposits or at least one large calcified deposit.

For immunofluorescent dual staining of microglia with TMEM119 and Iba1, paraffin sections were deparaffinized, rehydrated, treated to 25 mM Tris pH 8, 5 mM EDTA pH 8 and 0.05% (w/v) SDS buffer antigen retrieval and blocked as above. Rabbit anti-TMEM119 (ab209064, Abcam, 1:100) was incubated for 2 h at RT. Following thorough washing, sections were incubated with anti-rabbit IgG-AF594 (A-11037, Thermo Fisher Scientific, 1:500) for 2 h at RT. Slides were thoroughly washed in PBS and then rabbit anti-Iba1 (1:500) was incubated overnight at 4°C. Following thorough washing, sections were incubated with anti-rabbit IgG-AF488 (A-11034, Thermo Fisher Scientific, 1:500) for 1 h at RT. Slides were washed and then cover-slipped with Fluoroshield™ DAPI mounting media (Sigma-Aldrich). Fluorescent imaging was performed at the Advanced Microscopy Facility of the Bosch Institute at the University of Sydney using a Zeiss LSM800 confocal laser scanning microscope using 40×Plan Apochromat NA=1.3 oil-immersion objective, or 63×Plan Apochromat NA=1.4 oil-immersion objective with 405, 488 and 561 nm lasers and appropriate filters (Carl Zeiss). The number of immunolabelled cells was counted using the Cell Counter plugin in ImageJ software (NIH, USA).

### RNA isolation, cDNA synthesis and qPCR analysis

Total RNA was prepared from snap frozen cerebella using TRI Reagent (Sigma-Aldrich) according to the manufacturer’s instructions. Purity and concentration of RNA was assessed using a Nanodrop-1000 spectrophotometer (Thermo Fisher Scientific).

To ensure that isolated total RNA was free from any DNA contaminants, 1 μg of cerebellar tissue RNA was incubated with 1 U of RQ1 RNase-free DNase (Promega) for 30 min at 37°C according to the manufacturer’s instructions. DNase-treated cerebellar tissue RNA was then reverse-transcribed using RevertAid First Strand cDNA synthesis kit (Thermo Fisher Scientific) according to the manufacturer’s instructions. The cDNA was diluted such that qPCR was performed on 10 ng of cerebellar cDNA. The qPCR was set up using SensiFAST™ SYBR® Lo-ROX (Meridian Bioscience) and 400 nM primer pairs and was performed with a 7500 Fast Real-Time PCR System (Thermo Fisher Scientific) using the ΔΔCt setting with the cycle program: 95°C for 2 min, then 40 cycles of 95°C for 5 sec then 60°C for 30 sec, followed by melt curve analysis. The C_T_ for each gene of interest was normalized to the C_T_ of the housekeeping gene 18S rRNA. Primer sequences: *Apoe* (forward: TGTGGGCCGTGCTGTTGGTC; reverse: GCCTGCTCCCAGGGTTGGTTG) (46), *Csf1* (forward: AGTATTGCCAAGGAGGTGTCAG; reverse: ATCTGGCATGAAGTCTCCATTT) (47), *Csf1r* (forward: CAGTTCAGAGTGATGTGTGGTC; reverse: CTTGTTGTTCACTAGGATGCCG) (47), *Csf2* (forward: CCAGCTCTGAATCCAGCTTCTC; reverse: TCTCTCGTTTGTCTTCCGCTGT) (48), *Cxcl10* (forward: AGAGACATCCCGAGCCAA; reverse: GATGAGGCAGAAAATGACGG), *Fn1* (forward: ACCGACAGTGGTGTGGTCTA; reverse: CACCATAAGTCTGGGTCACG) (49), total *Ifna* (forward: GTGACCTTCCTCAGACTCATAAC; reverse: CAAAGTCCTTCCTGTCCTTCA), transgenic *Ifna* (forward: CAATGTGCTGGGAAGACTGA; reverse: CTGCATTCTAGTTGTGGTTTGTC), *Ifng* (forward: CGGCACAGTCATTGAAAGCCTA; reverse: GTTGCTGATGGCCTGATTGTC) (50), *Il1b* (forward: TGGACCTTCCAGGATGAGGACA; reverse: GTTCATCTCGGAGCCTGTAGTG) (51), total *Il6* (forward: CAAAGCCAGAGTCCTTCAGA; reverse: GATGGTCTTGGTCCTTAGCC) (52), transgenic *Il6* (forward: TCACTTTGAGATCTACTCGGCA; reverse: CTGCATTCTAGTTGTGGTTTGTC), *Il12b* (IL-12 p40) (forward: ATGAGAACTACAGCACCAGCTTC; reverse: ACTTGAGGGAGAAGTAGGAATGG) (53), *Il18* (forward: GTGAACCCCAGACCAGACTG; reverse: CCTGGAACACGTTTCTGAAAGA) (54), *Il34* (forward: CTTTGGGAAACGAGAATTTGGAGA; reverse: GCAATCCTGTAGTTGATGGGGAAG) (55), *Isg15* (forward: GAGCTAGAGCCTGCAGCAAT; reverse: TTCTGGGCAATCTGCTTCTT) (56), *Mx1* (forward: TCTGAGGAGAGCCAGACGAT; reverse: ACTCTGGTCCCCAATGACAG) (56), *Oasl2* (forward: GGATGCCTGGGAGAGAATCG; reverse: TCGCCTGCTCTTCGAAAC) (56), *Serpina3n* (forward: GGGATGATCAAGGAACTGGTCT; reverse: CCGCGTAGAACTCAGACTTGAA) (57), *Socs3* (forward: GGAACTTGTTTGCGCTTTGATT; reverse: TCACACACCCTTTTCTCTTCCAT) (58), *Tgfb* (forward: GGAGAGCCCTGGATACCAAC; reverse: CAACCCAGGTCCTTCCTAAA) (59), *Tnf* (forward: GGTGCCTATGTCTCAGCCTCTT; reverse: GCCATAGAACTGATGAGAGGGAG) (51), *Vegfa* (forward: GTTCATGGATGTCTACCAGCGAAG; reverse: GAAGATGTACTCTATCTCGTCGGG) (60) and *18S* (forward: CACGGCCGGTACAGTGAAAC; reverse: AGAGGAGCGAGCGACCAA) (61).

### Statistics

For survival analysis, pairwise comparison of log-rank test was performed using the ‘survminer’ package in R (62). For clinical score ordinal data with repeated measures, except for one timepoint in one untreated GFAP-IL6 mouse, untreated and PLX5622-treated WT or GFAP-IL6 mice did not have a clinical score above zero and could not be modelled. Therefore, comparison of clinical scores of untreated and PLX5622-treated GFAP-IFN mice was performed with a cumulative link mixed model fitted with the Laplace approximation with the ‘ordinal’ package in R (63), with model: ‘clinical score ~ genotype/treatment + time + 1|mouseID’ and p-values were adjusted using the Bonferroni method. For weight analysis, statistical analyses of repeated measures with missing data were performed with linear mixed-effects model using the ‘lme4’ package in R (64) with model: ‘weight ~ genotype/treatment + 1|time + 1|mouseID’. Pairwise comparisons with Tukey p-value adjustment were performed using the ‘emmeans’ package in R (65). For analysis of calcification scoring, test for associations with control or PLX5622 treatment within and between genotypes for calcification was performed using a chisq_test function (66) and pairwise OrdinalIndependence function (67) for pairwise comparisons with p-values adjusted using the Benjamini-Hochberg method. All other statistical calculations were performed using Prism version 9 (GraphPad Software) and the specific statistical tests used to determine significance are indicated in the figure legends. For all data comparisons a p-value <0.05 was considered statistically significant.

## Results

### The dynamics of microglia repopulation are uniquely modulated following PLX5622 treatment in GFAP-IL6 versus GFAP-IFN brain

**Figure 1:**
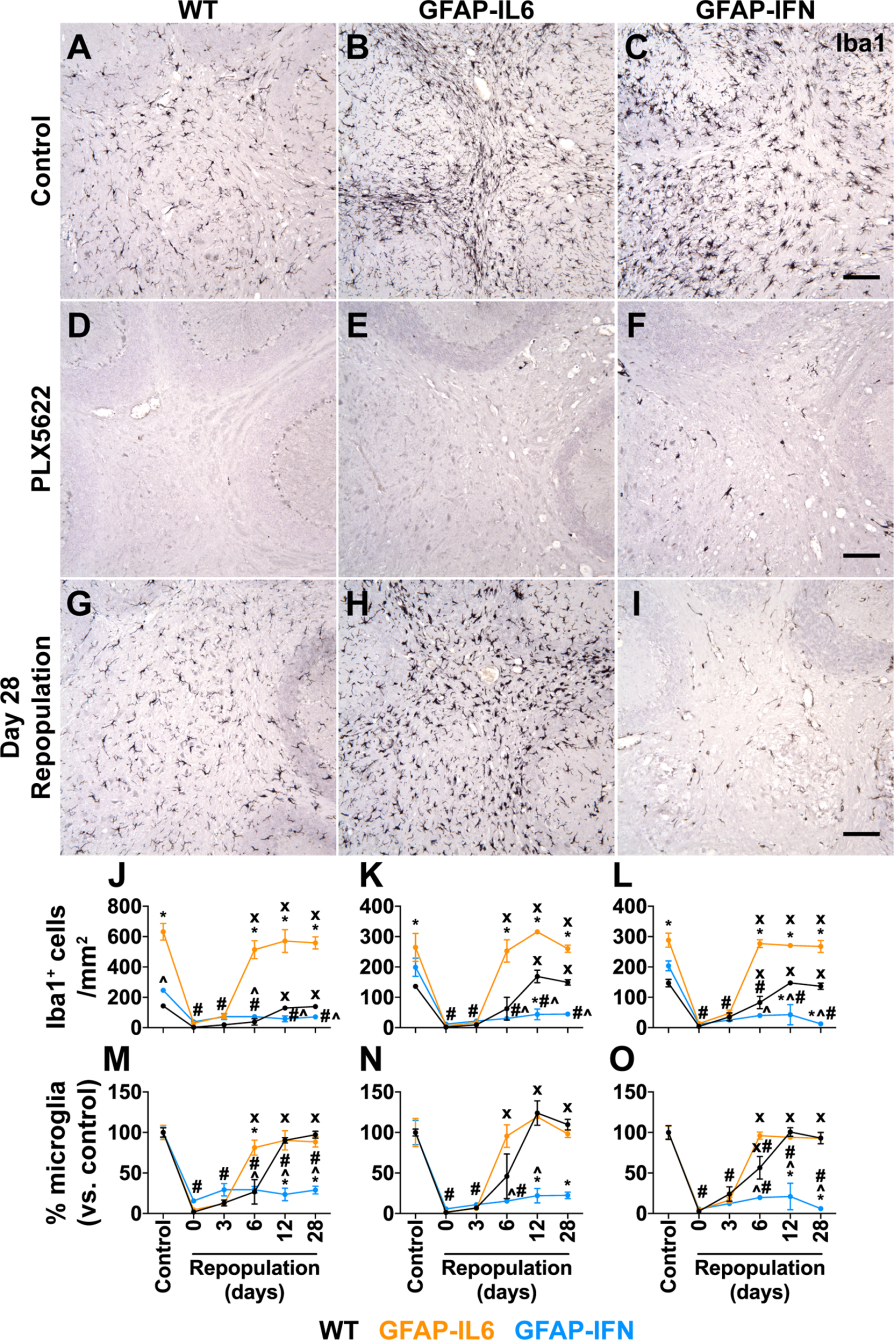
Microglia are ablated from the brain of PLX5622-treated mice and have unique repopulation properties in response to IL-6 versus IFN-α. **(A–I)** Immunohistochemistry for Iba1 performed on brain sections from mice given control or PLX5622 diet at 1-month-old for 14 days, or at different timepoints following removal of PLX5622. Scale bars, 100 μm. Representative images from the cerebellum shown. **(J–L)** Quantification of the number of Iba1^+^ cells in the **(J)** cerebellum, **(K)** cortex and **(L)** hippocampus of mice following withdrawal of PLX5622 for 0, 3, 6, 12 and 28 days. **(M–O)** Percentage (versus control) of the number of Iba1^+^ cells in the **(M)** cerebellum, **(N)** cortex and **(O)** hippocampus of mice following withdrawal of PLX5622 for 0, 3, 6, 12 and 28 days. n=3-6 mice/group. For **(J–O)**, graphs show mean ± SEM. *p<0.05 compared with WT of same diet and timepoint; ^p<0.05 compared with GFAP-IL6 of same diet and timepoint; ^#^p<0.05 compared with untreated control of same genotype; x, p<0.05 compared with PLX5622 day 0 of same genotype using two-way ANOVA with Tukey’s post-test.

PLX5622 rapidly ablates microglia from the brain and the few remaining microglia rapidly proliferate and repopulate the depleted niche following termination of PLX5622 treatment (22). To determine the efficiency of depletion and the repopulation capacity of microglia in GFAP-IL6 versus GFAP-IFN mice, WT, GFAP-IL6 and GFAP-IFN mice were treated with PLX5622 for 14 days and then PLX5622 was withdrawn for 0, 3, 6, 12 or 28 days. In all three brain regions analyzed, microglia were effectively and similarly depleted following PLX5622 treatment for 14 days (**Figures 1A–L**), with more than an 85% reduction of microglial cell numbers compared with vehicle-treated controls (**Figures 1M–O**). For all three genotypes, there were no significant differences in the number of CSF1R inhibition-resistant microglia in the cerebellum, cortex and hippocampus following PLX5622 treatment, indicating that CSF1R antagonist-mediated depletion was substantial, widespread and not affected by chronic production of IL-6 or IFN-α.

By contrast, following cessation of PLX5622 treatment, microglia had divergent abilities to repopulate the brain depending on the cytokine milieu. Microglia in the brain of WT mice repopulated all three brain regions analyzed and at 12 days following removal of PLX5622 treatment had comparable numbers to untreated WT mice (**Figures 1M–O**). Although there were significantly more numbers of microglia in the brain of untreated GFAP-IL6 mice compared with WT and GFAP-IFN mice (**Figures 1J–L**), microglia in GFAP-IL6 mice rapidly recovered from PLX5622-induced depletion and had comparable numbers at day 6 to untreated GFAP-IL6 mice (**Figures 1M–O**). However, microglia in GFAP-IFN mice did not repopulate the brain. The number of these cells 28 days after removal of PLX5622 remained more than 70% lower compared with untreated GFAP-IFN mice. Dual immunofluorescence demonstrated that virtually all Iba1^+^ cells in the brain of WT and GFAP-IL6 mice following PLX5622 withdrawal were also positive for the microglia-specific marker TMEM119 (**Supplementary Figure 1**), indicating that there was negligible recruitment of peripheral monocytes or macrophages to the depleted niche and that the brains of these animals were repopulated by microglia.

**Figure 2:**
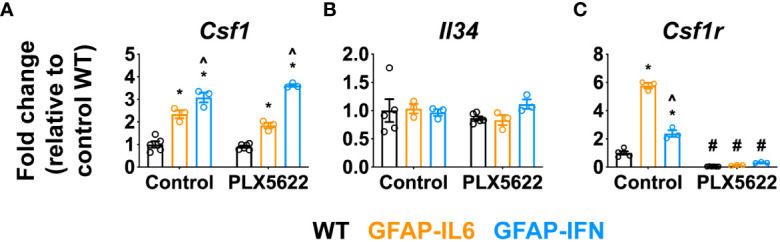
*Csf1* and *Il34* expression does not play a major role in the repopulation capacity of PLX5622-resistant microglia in the CNS of GFAP-IL6 versus GFAP-IFN mice. **(A–C)** qPCR of microglia growth factors **(A)** *Csf1* and **(B)** *Il34* and their receptor **(C)** *Csf1r* in the cerebellum of 1-month-old WT, GFAP-IL6 and GFAP-IFN mice treated with control or PLX5622 diet for 14 days. n=3-6 mice/group. Graphs show individual values per mouse and mean ± SEM. *p<0.05 compared with WT of same condition; ^p<0.05 compared with GFAP-IL6 of same condition; ^#^p<0.05 compared with untreated control of same genotype using two-way ANOVA with Tukey’s post-test.

Proliferation, differentiation and survival of microglia are critically regulated by CSF1R signaling, triggered by the binding of CSF1 or IL-34 (18). In order to determine whether the cytokine environments driven by chronic IL-6 or IFN-α production regulated the expression of these key microglia growth factors, we examined expression of *Csf1*, *Il34* and *Csf1r* in the cerebellum (**Figures 2A–C**). Compared with WT mice, expression of *Csf1* was significantly upregulated in GFAP-IL6 and GFAP-IFN mice independent of the PLX5622 treatment (**Figure 2A**). Expression of *Csf1* was further increased in GFAP-IFN mice compared with GFAP-IL6 mice. On the other hand, all three genotypes, regardless of treatment, had comparable levels of *Il34* mRNA (**Figure 2B**). In untreated mice the expression of *Csf1r* paralleled the abundance of microglia in the cerebellum (**Figure 2C**). Expression was increased 6- and 2.4-fold in the cerebellum of non-treated GFAP-IL6 and GFAP-IFN mice respectively compared with WT, while *Csf1r* mRNA levels were markedly reduced in PLX5622-treated mice from all three genotypes (**Figure 2C**). Taken together, these findings suggest that the ability of microglia to recover from acute ablation is not altered by PLX5622-induced changes in expression of the growth factors CSF1 or IL-34 in the brain of GFAP-IL6 and GFAP-IFN mice. Instead, the ability of microglia to repopulate is divergently altered by the IL-6 versus IFN-α-driven cytokine environments.

### Long-term PLX5622 treatment of GFAP-IFN but not GFAP-IL6 mice is deleterious and results in reduced survival

**Figure 3:**
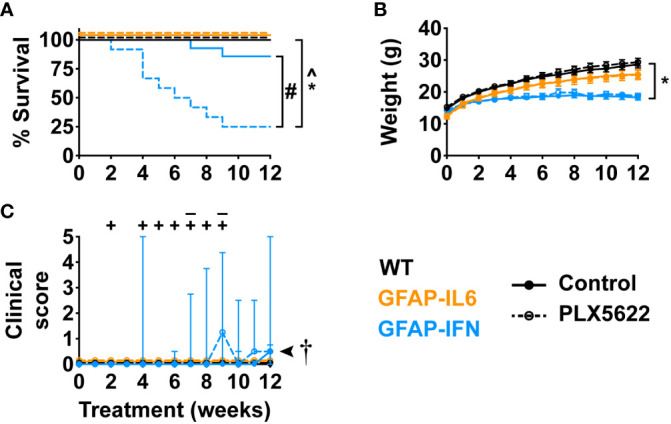
PLX5622 treatment increases the susceptibility of GFAP-IFN mice to seizures and death. **(A)** Survival, **(B)** weight and **(C)** clinical score of 1-month-old mice treated with control or PLX5622 diet for 12 weeks. Solid lines represent control diet-fed mice and dashed lines represent PLX5622 diet-fed mice. In **(C)**, the ‘–’ symbols indicate when untreated GFAP-IFN mice died and ‘+’ symbols indicate when PLX5622-treated GFAP-IFN animals died. n=12-24 mice/group. For **(B)**, graph shows mean ± SEM, for **(C)**, graph shows median + interquartile range. Significance of survival between genotypes and treatment groups was calculated by the log-rank test with Benjamini-Hochberg post-test. Significance of weight between genotypes and treatment groups was calculated with linear mixed-effects models and p-values adjusted with Tukey’s post-test. Significance of clinical scores between untreated and PLX5622-treated GFAP-IFN mice was calculated with cumulative link mixed models with p-values adjusted using the Bonferroni method. *p<0.05 compared with WT of same diet; ^p<0.05 compared with GFAP-IL6 of same diet; ^#^p<0.05 compared with untreated control of same genotype; †, the significance of the clinical scores of PLX5622-treated GFAP-IFN mice compared with untreated GFAP-IFN mice was p<0.05.

Next, in order to elucidate the effect of CSF1R inhibition on the progression of neuroinflammatory disease exhibited by GFAP-IL6 and GFAP-IFN mice, we treated 1-month-old mice with control or PLX5622 diet for 12 weeks and monitored their clinical phenotype. Compared with untreated and PLX5622-treated WT and GFAP-IL6 and untreated GFAP-IFN mice, PLX5622-treated GFAP-IFN mice progressively died and had a significantly reduced median survival time of 6.5 weeks of treatment (**Figure 3A**). Of the GFAP-IFN mice that did not survive 12 weeks of PLX5622 treatment, most were found dead, however, some were observed having seizures requiring euthanasia (**Figure 3C**). While non-treated and PLX5622-treated WT and GFAP-IL6 mice continually gained weight throughout the experiment and had similar weights, both untreated and PLX5622-treated GFAP-IFN mice stopped gaining weight from five to six weeks of treatment and were significantly lighter than the untreated and PLX5622-treated WT mice across the experiment (**Figure 3B**). Increased seizure frequency and lethality in PLX5622-treated GFAP-IFN mice did not correspond to reduced weight compared with untreated GFAP-IFN mice (**Figure 3B**), but resulted in a significantly increased disease score (**Figure 3C**).

### Both PLX5622-treated GFAP-IL6 and GFAP-IFN mice exhibit exaggerated features of disease with severe calcification

**Figure 4:**
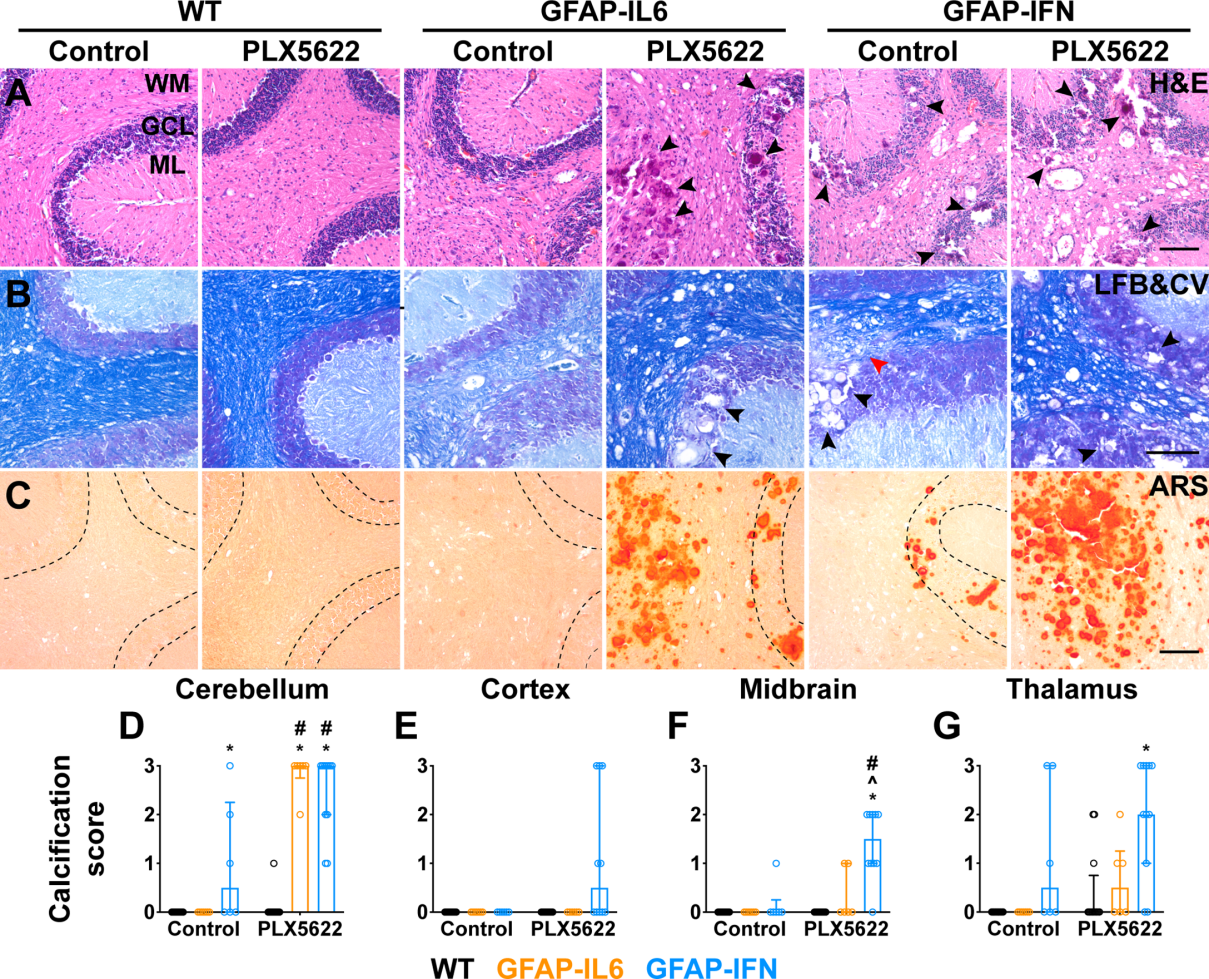
Exaggerated gross features of disease, with severe calcification, in both GFAP-IL6 and GFAP-IFN mice treated with PLX5622. **(A-C)** Representative images of the cerebellum of mice treated with control or PLX5622 diet for 12 weeks. **(A)** Hematoxylin and eosin (H&E), **(B)** Luxol fast blue and cresyl violet (LFB&CV) and **(C)** Alizarin red S (ARS) stains. WM: white matter, GCL: granule cell layer and ML: molecular layer. Black arrowheads indicate calcified deposits and red arrowhead indicates area with focal demyelination. Scale bars, 100 μm. **(D-G)** Calcification scores in the **(D)** cerebellum, **(E)** cortex, **(F)** midbrain and **(G)** thalamus. n=6-12 mice/group. Graphs show individual values per mouse and median + interquartile range. Significance of associations with control or PLX5622 treatment within and between genotypes for calcification scores was calculated with modified chi squared tests with p-values adjusted with Benjamini-Hochberg post-test. *p<0.05 compared with WT of same diet; ^p<0.05 compared with GFAP-IL6 of same diet; ^#^p<0.05 compared with untreated control of same genotype.

The reduced survival and more severe disease in PLX5622-treated GFAP-IFN mice led us to next examine gross histopathological changes in the brain of these mice. The cerebellum of both untreated and PLX5622-treated WT mice showed no overt pathology (**Figures 4A–C**). By contrast, H&E-stained sections of the cerebellum of non-treated GFAP-IL6 mice showed increased vacuolation and demyelination of the cerebellar white matter, vascular abnormalities, including enlarged blood vessels, as well as perivascular leukocytes (**Figures 4A, B**). While PLX5622-treated GFAP-IL6 mice had comparable myelination to WT mice (**Figure 4B**), PLX5622 treatment exaggerated other pathological changes in the cerebellum of GFAP-IL6 mice. In particular, the cerebellum of PLX5622-treated GFAP-IL6 mice contained extensive calcified deposits predominantly in the granule cell layer and white matter tracts (**Figures 4A, C**). By contrast, GFAP-IFN mice showed cerebellar white matter vacuolation which were associated with focal demyelination, enlarged blood vessels with thickened walls, as well as perivascular leukocytes, which were not markedly different between untreated and treated GFAP-IFN mice (**Figures 4A, B**). There was no evidence of herniation of the brain of PLX5622-treated GFAP-IFN mice, since necrosis or compressed ventricles were absent from mice found dead. While calcification was also seen in the cerebella of GFAP-IFN mice, this was enhanced by PLX5622 treatment. Further, scoring of the severity of calcification demonstrated that calcification in PLX5622-treated GFAP-IL6 mice was largely limited to the cerebellum, while PLX5622-treated GFAP-IFN mice had significant, extensive deposition throughout the brain (**Figures 4D–G**). Taken together, these observations indicate that PLX5622 treatment results in exaggerated gross histopathological changes in the brain of both GFAP-IL6 and GFAP-IFN mice, in particular prominent calcification.

### PLX5622-treated GFAP-IL6 and GFAP-IFN mice do not exhibit exaggerated astrocytosis, vasculopathy, neurodegeneration or immune cell infiltration

**Figure 5:**
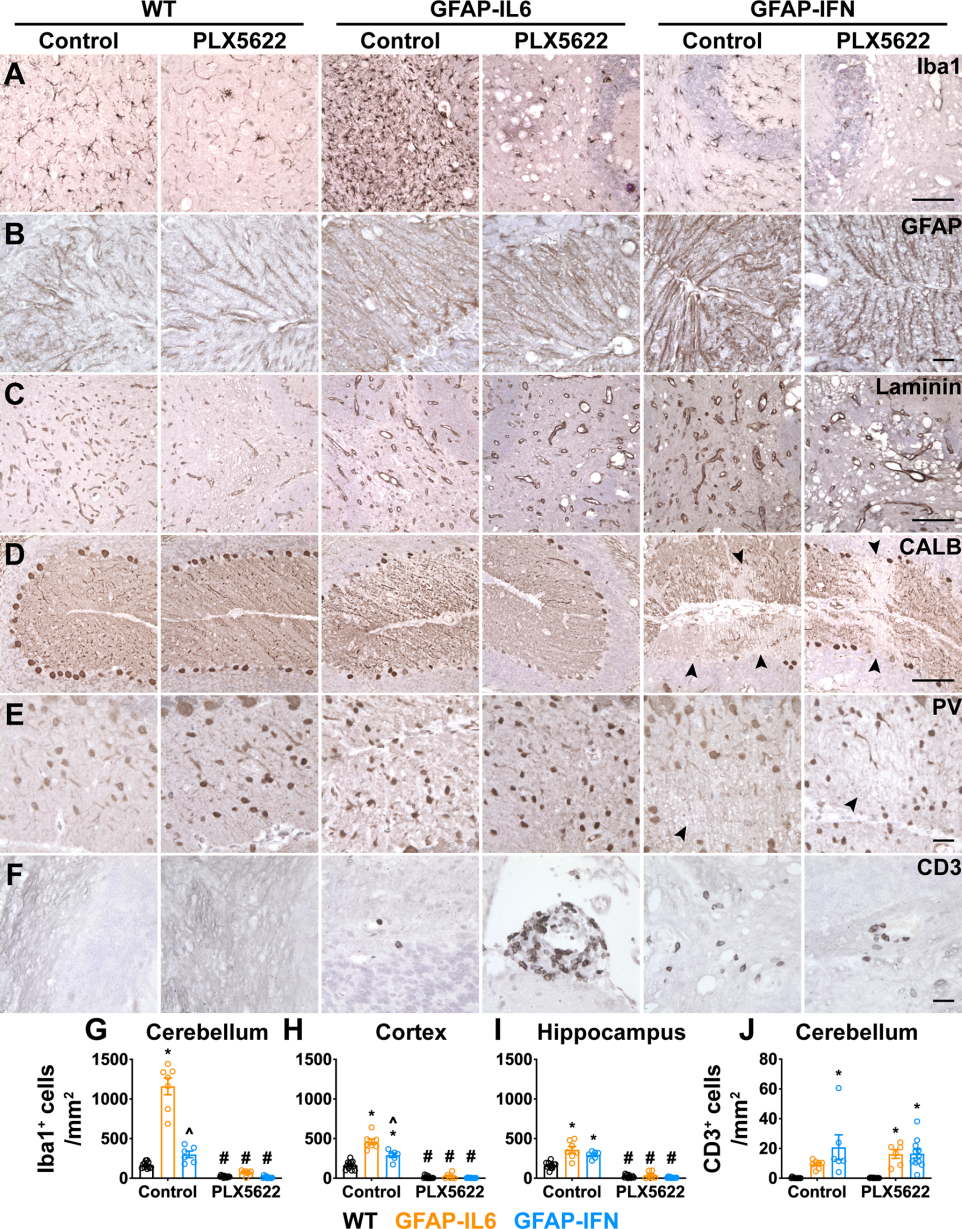
PLX5622 treatment of GFAP-IL6 and GFAP-IFN mice does not overtly exaggerate astrocytosis, vasculopathy, neurodegeneration or immune cell infiltration in the brain. **(A–F)** Representative images of the cerebellum of mice treated with control or PLX5622 diet for 12 weeks. **(A)** Iba1, **(B)** GFAP, **(C)** laminin, **(D)** calbindin (CALB), **(E)** parvalbumin (PV) and **(F)** CD3 in the cerebellum. Black arrowheads indicate areas of neuronal cell loss. Scale bars for (A, C, D), 100 μm. Scale bars for (B, E, F), 20 μm. **(G–I)** Quantification of the total number of Iba1^+^ microglia per mm^2^ in the **(G)** cerebellum, **(H)** cortex and **(I)** hippocampus. **(J)** Quantification of the number of parenchymal, perivascular and meningeal CD3^+^ T cells per mm^2^ in the cerebellum. n=6-12 mice/group. Graphs show individual values per mouse and mean ± SEM. *p<0.05 compared with WT of same condition; ^p<0.05 compared with GFAP-IL6 of same condition; ^#^p<0.05 compared with untreated control of same genotype using two-way ANOVA with Tukey’s post-test.

We next performed more detailed histopathological investigation of the brain of PLX5622-treated GFAP-IL6 and GFAP-IFN mice by performing immunohistochemistry for glial cells, blood vessels, neurons and infiltrating leukocytes (**Figure 5**, **Supplementary Figure 2**). Expectedly, Iba1 staining (**Figure 5A**, **Supplementary Figure 2A**) demonstrated significant depletion of microglia in the brains of PLX5622-treated mice from all three genotypes (**Figures 5G–I**). Compared with WT mice, GFAP staining revealed strong astrogliosis in the cerebella of GFAP-IL6 and GFAP-IFN mice that was comparable in untreated and PLX5622-treated animals (**Figure 5B**, **Supplementary Figure 2B**). In particular, astrocyte processes were associated with vacuolation in the molecular layer and white matter. Similar findings were also seen in the cerebrum (**Supplementary Figure 2C**). Compared with WT mice, analysis of vascular changes by laminin staining confirmed that both non-treated GFAP-IL6 and GFAP-IFN mice had vascular abnormalities, including increased numbers of enlarged, dilated blood vessels with thick vessel walls, as well as pathologically enlarged vessels with thin vessel walls (**Figure 5C**, **Supplementary Figure 2D**). Some of the vacuolation observed above in H&E-stained sections was not associated with laminin staining, indicative of tissue destruction. The vasculopathy in PLX5622-treated GFAP-IL6 and GFAP-IFN mice was comparable to untreated animals. In the cerebellum of untreated and PLX5622-treated WT and GFAP-IL6 mice, the somata and dendrites of Purkinje neurons were both NF-200- (**Supplementary Figure 2E**) and calbindin-positive (**Figure 5D**), while molecular layer interneurons were parvalbumin-positive (**Figure 5E**). The number of Purkinje neurons and molecular layer interneurons in these mice did not appear to be affected by PLX5622 treatment (**Figures 5D, E**). By contrast, there was loss of NF-200 and calbindin staining of Purkinje cells in the outer folia of the cerebellum, with focal loss in regions in close proximity to calcifications and tissue destruction in both untreated and PLX5622-treated GFAP-IFN mice (**Supplementary Figures 2E, F**, **Figure 5D**). In line with more extensive calcification and tissue destruction, PLX5622-treated GFAP-IFN mice showed more widespread loss of Purkinje cells. Despite a significant focal loss of Purkinje cells, when larger areas of the Purkinje cell layer (PCL) were analyzed, these changes were not pronounced and were comparable between untreated and PLX5622-treated GFAP-IFN mice (**Supplementary Figure 2G**), indicating that enhanced neuronal loss was unlikely to contribute to the exaggerated clinical disease of PLX5622-treated GFAP-IFN mice. In addition, there was also loss of parvalbumin-positive interneurons in the molecular layer of untreated and PLX5622-treated GFAP-IFN mice in areas of severe calcification and vacuolation (**Figure 5E**). Finally, infiltrating CD3^+^ T cells were barely detected in the cerebellum of untreated and PLX5622-treated WT mice (**Figures 5F, J**). PLX5622-treated GFAP-IL6 mice had significantly increased numbers of T cells compared with WT mice, with a significant proportion of T cells observed in the meninges, however, these numbers were comparable to non-treated GFAP-IL6 mice. Similarly, although both untreated and PLX5622-treated GFAP-IFN mice had significantly increased numbers of perivascular and parenchymal T cells in the cerebellum compared with WT mice, PLX5622 treatment did not significantly alter the number of infiltrating T cells.

Overall, while PLX5622 treatment and microglia ablation of GFAP-IL6 and GFAP-IFN mice resulted in enhanced tissue destruction and calcification, as well as increased incidence of death in GFAP-IFN mice, the histopathological changes demonstrate there were no overt cellular changes consistent with exaggerated astrocytopathy, vasculopathy, neurodegeneration or immune cell infiltration.

### PLX5622-treated GFAP-IL6 and GFAP-IFN mice do not exhibit increased expression of IFN-α or IL-6 or their regulated genes

**Figure 6:**
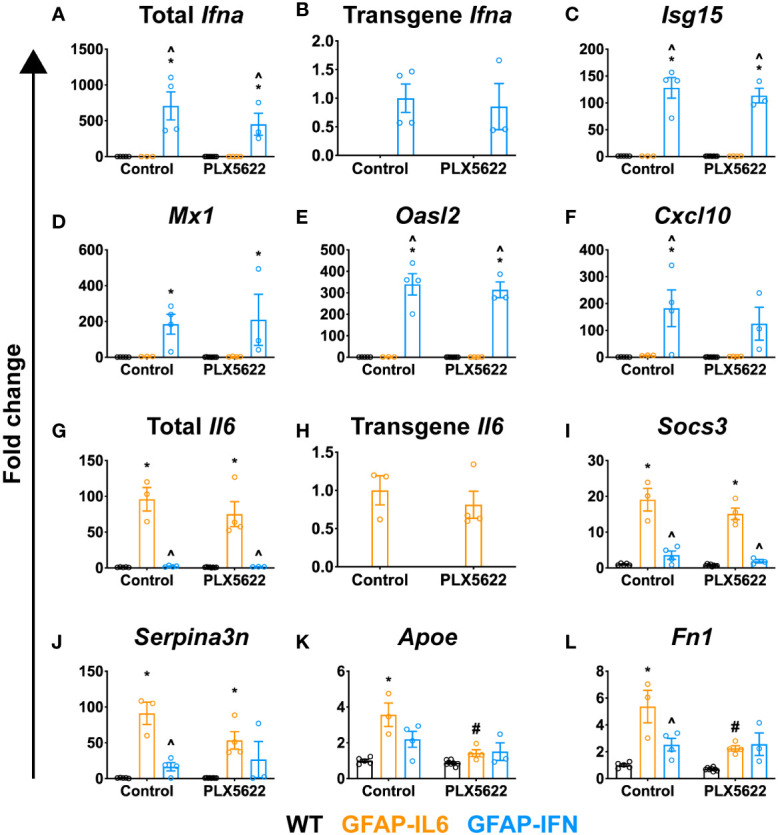
PLX5622-mediated ablation of microglia in the brain of GFAP-IL6 and GFAP-IFN mice does not alter the expression of IL-6 or IFN-α or their regulated genes. **(A–L)** qPCR of selected IL-6- and IFN-α-regulated genes in the cerebellum of WT, GFAP-IL6 and GFAP-IFN mice treated with control or PLX5622 diet for 12 weeks. **(A–F)** Fold change of IFN-α-regulated genes: **(A)** total *Ifna*, **(B)** transgene *Ifna*, **(C)** *Isg15*, **(D)** *Mx1*, **(E)** *Oasl2* and **(F)** *Cxcl10*. **(G–L)** Fold change of IL-6-regulated genes: **(G)** total *Il6*, **(H)** transgene *Il6*, **(I)** *Socs3*, **(J)** *Serpina3n*, **(K)** *Apoe* and **(L)** *Fn1*. In **(B)**, fold change is relative to untreated GFAP-IFN mice; in **(H)**, fold change is relative to untreated GFAP-IL6 mice; and in **(A, C–G, I–L)**, fold change is relative to untreated WT mice. n=3-8 mice/group. Graphs show individual values per mouse and mean ± SEM. *p<0.05 compared with WT of same condition; ^p<0.05 compared with GFAP-IL6 of same condition; ^#^p<0.05 compared with untreated control of same genotype using two-way ANOVA with Tukey’s post-test.

Cerebral calcifications are a cardinal feature in cerebral interferonopathies, a group of diseases driven by chronically exaggerated cerebral IFN-α production (68, 69). We surmised that elevated IFN-α production following PLX5622 treatment might account for the increased calcification found in treated GFAP-IL6 and GFAP-IFN mice. Thus, we next examined cerebellar expression of IFN-α and IFN-regulated genes, and IL-6- and IL-6-regulated genes. Expression of total *Ifna* and transgenic *Ifna* was detected only in the cerebellum of GFAP-IFN mice and was not significantly altered by PLX5622 treatment (**Figures 6A, B**). Importantly, expression of *Ifna* in the cerebellum of PLX5622-treated GFAP-IL6 mice was very low and comparable to WT mice (**Figure 6A**). Expression of IFN-regulated genes, such as *Isg15* (**Figure 6C**), *Mx1* (**Figure 6D**), *Oasl2* (**Figure 6E**) and *Cxcl10* (**Figure 6F**), was upregulated to a similar degree in the cerebellum of both untreated and PLX5622-treated GFAP-IFN mice compared with WT and GFAP-IL6 mice. Similarly, expression of total *Il6* and transgenic *Il6* was observed only in the cerebellum of GFAP-IL6 mice and was not significantly altered by PLX5622 treatment (**Figures 6G, H**). Expression of IL-6-regulated genes, such as *Socs3* (**Figure 6I**), *Serpina3n* (**Figure 6J**), *Apoe* (**Figure 6K**) and *Fn1* (**Figure 6L**), was significantly upregulated in non-treated GFAP-IL6 mice compared with WT and GFAP-IFN mice. The expression of these genes was not altered by PLX5622 treatment of GFAP-IL6 mice, with the exception of *Apoe* and *Fn1*, genes strongly expressed by microglia in response to IL-6 (44), which were reduced (**Figures 6K, L**). Taken together, these findings indicate that PLX5622 treatment and microglial cell ablation did not induce exaggerated expression of IL-6, IFN-α, or their downstream regulated genes.

### PLX5622-treated GFAP-IL6 and GFAP-IFN mice do not exhibit increased expression of cytokine genes

**Figure 7:**
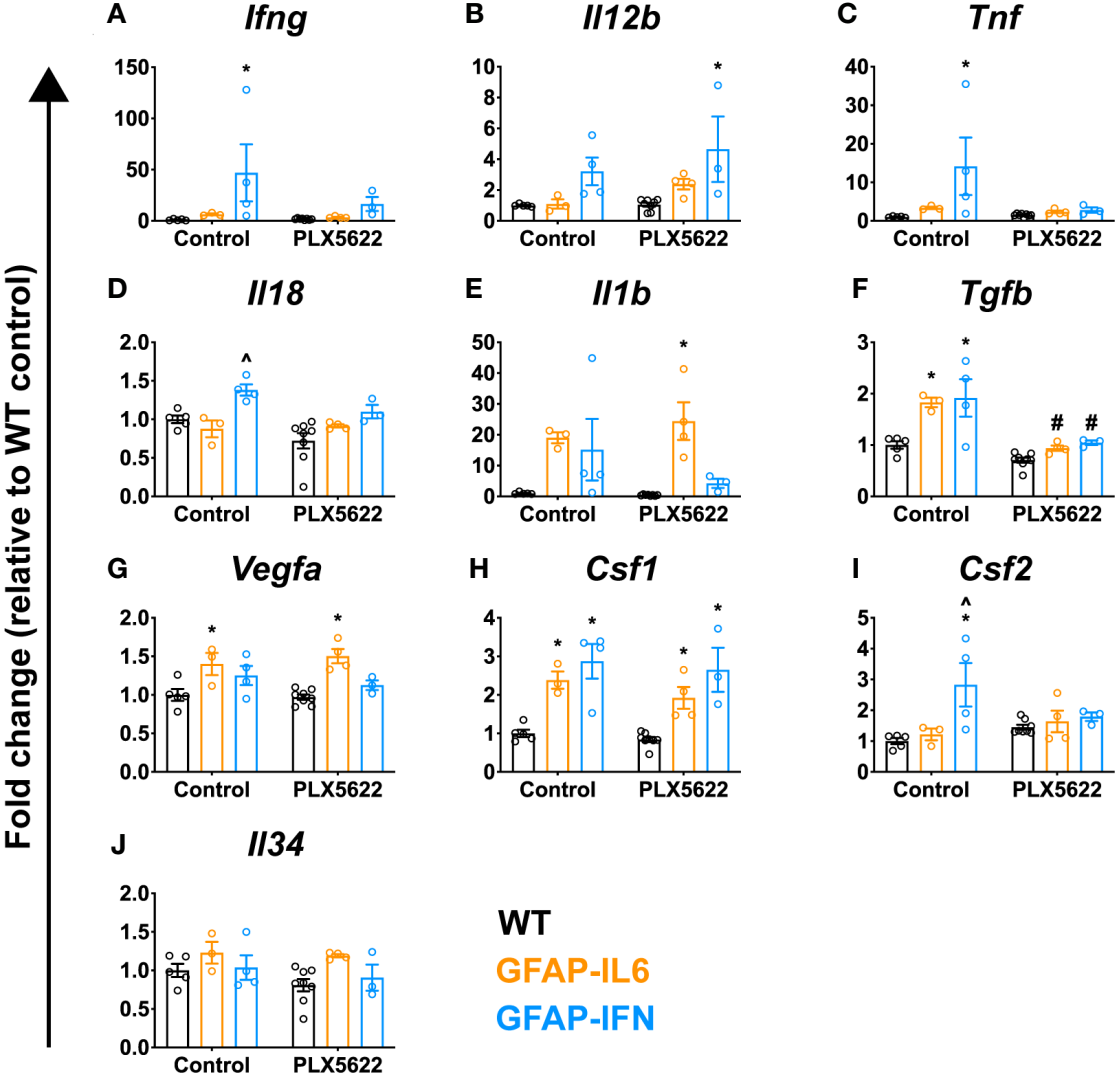
PLX5622-mediated ablation of microglia in the brain of GFAP-IL6 and GFAP-IFN mice does not result in enhanced cytokine gene expression. **(A–J)** qPCR of selected cytokine genes in the cerebellum of WT, GFAP-IL6 and GFAP-IFN mice treated with control or PLX5622 diet for 12 weeks. Fold change of the cytokine genes **(A)** *Ifng*, **(B)** *Il12b* (IL-12 p40), **(C)** *Tnf*, **(D)** *Il18*, **(E)** *Il1b*, **(F)** *Tgfb*, **(G)** *Vegfa*, **(H)** *Csf1*, **(I)** *Csf2* and **(J)** *Il34*. n=3-8 mice/group. Graphs show individual values per mouse and mean ± SEM. *p<0.05 compared with WT of same condition; ^p<0.05 compared with GFAP-IL6 of same condition; ^#^p<0.05 compared with untreated control of same genotype using two-way ANOVA with Tukey’s post-test.

Although PLX5622 treatment and microglia ablation does not induce an exaggerated IL-6- or IFN-α-mediated response, there are additional cytokines which can drive the deposition of calcium in the brain. For example, mice with CNS-targeted production of IL-12 develop severe neuroinflammatory disease and cerebellar calcifications *via* the induction of IFN-γ (70, 71). Similarly, adeno-associated virus (AAV)-driven overproduction of IFN-γ in the CNS of mice also results in progressive basal ganglia calcification (72). Compared with untreated WT mice, *Ifng* expression was significantly increased in untreated GFAP-IFN mice (**Figure 7A**). In PLX5622-treated GFAP-IFN mice, there was a non-significant decrease in the levels of *Ifng* mRNA compared with untreated mice. Similarly, compared with WT mice, levels of *Il12b* (IL-12 p40) mRNA were increased to comparable levels in untreated and PLX5622-treated GFAP-IFN mice (**Figure 7B**). Untreated and treated GFAP-IL6 mice showed mRNA levels for *Ifng* and *Il12b* comparable to WT mice. These findings demonstrate that the increased calcification in brains from PLX5622-treated GFAP-IL6 and GFAP-IFN mice did not correlate with the expression of these cytokines.

We therefore expanded our characterization of the neuroinflammation in these animals. There was elevated expression of *Tnf* and *Il18* in the cerebellum of untreated GFAP-IFN mice compared with WT and GFAP-IL6 mice (**Figures 7C, D**). Following microglia depletion, levels of *Tnf* and *Il18* mRNA decreased in GFAP-IFN mice to those seen in treated WT and GFAP-IL6 mice. Expression of *Il1b* and *Tgfb* was increased similarly in untreated GFAP-IL6 and GFAP-IFN mice compared with WT (**Figures 7E, F**). While *Il1b* mRNA levels were not significantly altered following microglia depletion (**Figure 7E**), PLX5622 treatment significantly reduced the levels of *Tgfb* expression in both GFAP-IL6 and GFAP-IFN mice compared with untreated mice (**Figure 7F**). Further, chronic expression of IL-6 in GFAP-IL6 mice increased expression of *Vegfa* (**Figure 7G**) and this increase was independent of the presence or absence of microglia. Finally, the expression of microglial cell growth factors in these animals (**Figures 7H–J**) was similar to that observed in mice treated with PLX5622 for 14 days (**Figure 2**). Compared with untreated WT mice, *Csf1* expression was comparably upregulated in untreated GFAP-IL6 and GFAP-IFN mice respectively and these changes were similar in PLX5622-treated mice (**Figure 7H**). While there was elevated expression of *Csf2* in untreated GFAP-IFN mice compared with WT and GFAP-IL6 mice, the levels were reduced following PLX5622 treatment (**Figure 7I**). All three genotypes, regardless of treatment, had comparable levels of *Il34* expression (**Figure 7J**). Taken together, these observations suggest that augmented expression of the inflammatory cytokines investigated here is not a causative factor for the exaggerated neuropathology observed in PLX5622-treated GFAP-IL6 and GFAP-IFN mice.

### Acute PLX5622 treatment recapitulates the molecular and clinical phenotype of chronic PLX5622-treated GFAP-IFN but not GFAP-IL6 mice

**Figure 8:**
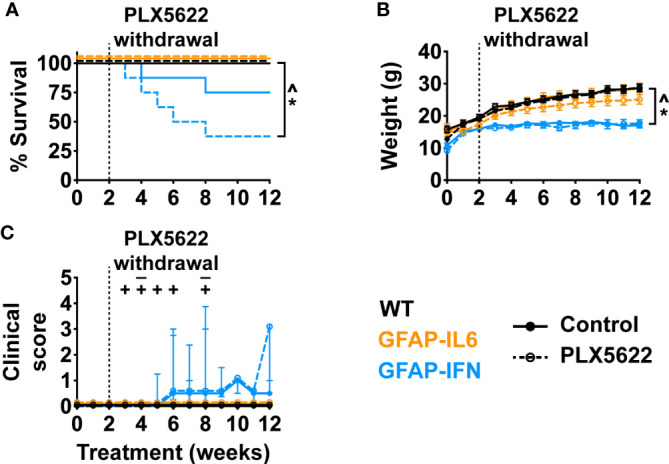
Ten weeks following PLX5622 withdrawal, GFAP-IFN mice recapitulate the clinical phenotype observed in chronic PLX5622-treated mice. **(A)** Survival, **(B)** weight and **(C)** clinical score of 1-month-old mice treated with control or PLX5622 diet for 2 weeks, followed by 10 weeks on control diet. Solid lines represent control diet-fed mice and dashed lines represent PLX5622 diet-fed mice. In **(C)**, the ‘–’ symbols indicate when untreated GFAP-IFN mice died and ‘+’ symbols indicate when PLX5622-treated GFAP-IFN animals died. n=7-16 mice/group. For **(B)**, graph shows mean ± SEM, for **(C)**, graph shows median + interquartile range. Significance of survival between genotypes and treatment groups was calculated by the log-rank test with Benjamini-Hochberg post-test. Significance of weight between genotypes and treatment groups was calculated with linear mixed-effects models and p-values adjusted with Tukey’s post-test. Significance of clinical scores between untreated and PLX5622-treated GFAP-IFN mice was calculated with cumulative link mixed models with p-values adjusted using the Bonferroni method. *p<0.05 compared with WT of same diet; ^p<0.05 compared with GFAP-IL6 of same diet; ^#^p<0.05 compared with untreated control of same genotype.

Loss of microglia resulted in increased morbidity and mortality in GFAP-IFN mice following chronic PLX5622 treatment. Since microglia repopulation of the brain of GFAP-IFN mice was also markedly reduced following acute PLX5622 treatment, we asked whether there was also an increase in morbidity and mortality in GFAP-IFN mice following transient treatment with PLX5622. To examine this notion, we treated 1-month-old mice with control or PLX5622 diet for 14 days, then removed PLX5622 treatment for 10 weeks, and monitored their clinical phenotype and examined the histopathological changes in the brain of these mice. Compared with untreated and acute PLX5622-treated WT and GFAP-IL6 mice, GFAP-IFN mice acutely treated with PLX5622 progressively died and had a significantly reduced median survival time of five weeks post cessation of treatment (**Figure 8A**). Similar to long-term treated animals (**Figure 3**), non-treated and short-term PLX5622-treated WT and GFAP-IL6 mice continually gained weight throughout the experiment and had similar weights, while both untreated and short-term PLX5622-treated GFAP-IFN mice stopped gaining weight from five to six weeks of treatment and were significantly lighter than the untreated and short-term PLX5622-treated WT and GFAP-IL6 mice (**Figure 8B**). Acute treatment of GFAP-IFN mice with PLX5622 did not result in greater loss of weight compared with untreated GFAP-IFN mice. While untreated and short-term PLX5622-treated WT and GFAP-IL6 mice had a median clinical score of zero during the course of the experiment, both untreated and short-term PLX5622-treated GFAP-IFN mice showed signs of mild ataxia beginning at six weeks (**Figure 8C**).

**Figure 9:**
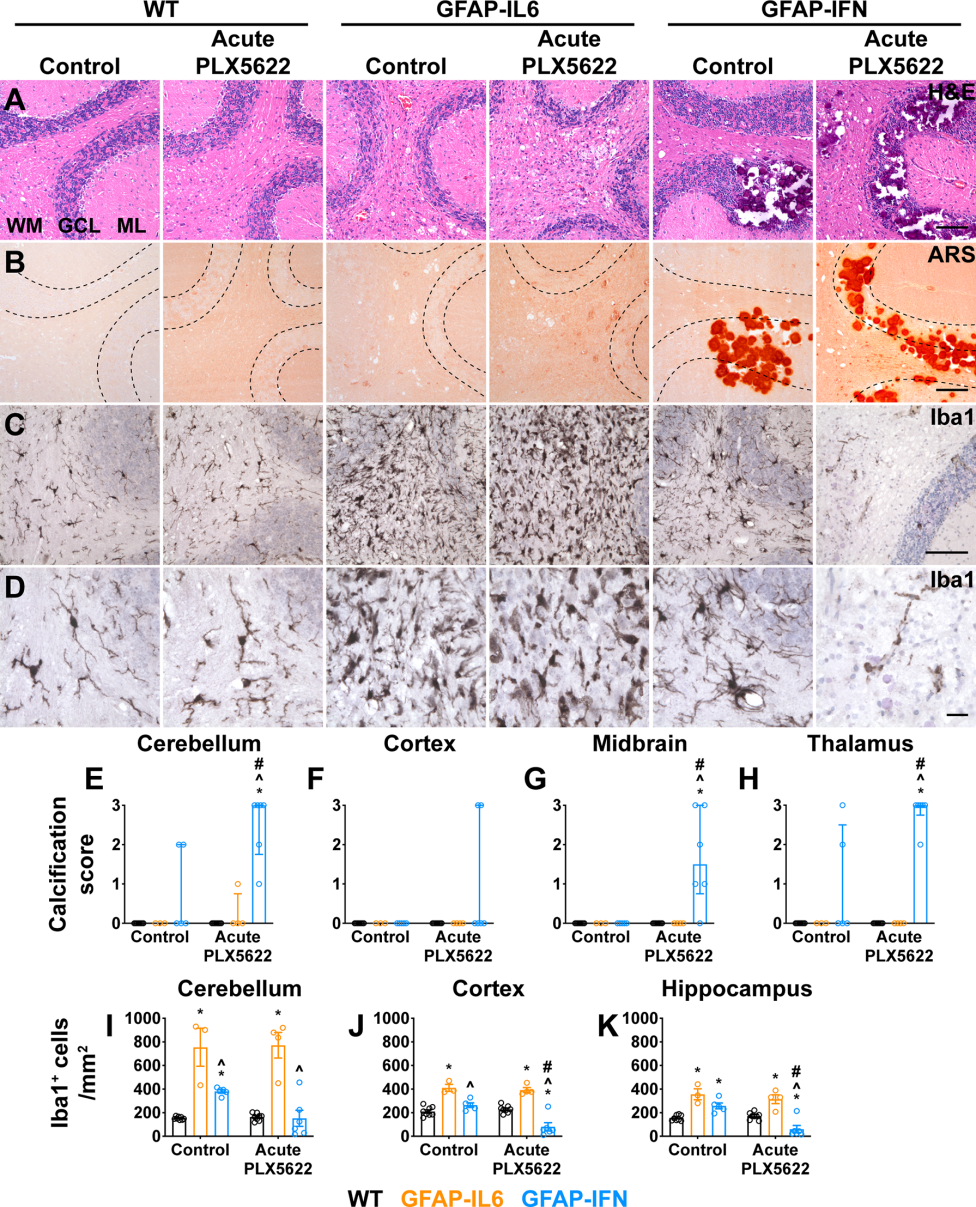
Ten weeks following PLX5622 withdrawal, GFAP-IFN mice but not GFAP-IL6 mice recapitulate the molecular phenotype observed in chronic PLX5622-treated mice. **(A–D)** Representative images of the cerebellum of mice treated with control or PLX5622 diet for 2 weeks, followed by 10 weeks on control diet. **(A)** H&E, **(B)** ARS and **(C, D)** Iba1. WM: white matter, GCL: granule cell layer and ML: molecular layer. Scale bars for **(A–C)**, 100 μm. Scale bars for **(D)**, 20 μm. **(E–H)** Calcification scores in the **(E)** cerebellum, **(F)** cortex, **(G)** midbrain and **(H)** thalamus. **(I–K)** Quantification of the total number of Iba1^+^ microglia per mm^2^ in the **(I)** cerebellum, **(J)** cortex and **(K)** hippocampus. n=3-8 mice/group. For **(E–H)**, graphs show individual values per mouse and median + interquartile range. For **(I–K)**, graphs show individual values per mouse and mean ± SEM. For **(E–H)**, significance was calculated with modified chi squared tests with p-values adjusted with Benjamini-Hochberg post-test. For **(I–K)**, significance was calculated using two-way ANOVA with Tukey’s post-test. *p<0.05 compared with WT of same diet; ^p<0.05 compared with GFAP-IL6 of same diet; ^#^p<0.05 compared with untreated control of same genotype.

Histological examination of the cerebellum of both untreated and short-term PLX5622-treated WT mice showed no overt changes (**Figure 9A**). By contrast, cerebella from both non-treated and short-term PLX5622-treated GFAP-IL6 and GFAP-IFN mice showed increased vacuolation of the cerebellar white matter, vascular aberrations, including enlarged blood vessels, as well as perivascular leukocytes. Importantly, GFAP-IL6 mice acutely treated with PLX5622 did not exhibit cerebellar calcifications, except for two small calcifications in the cerebellum of one mouse (**Figures 9A, B, E**). On the other hand, while there were small calcifications in the cerebellar granule cell layer and thalamus of non-treated GFAP-IFN mice, GFAP-IFN mice acutely treated with PLX5622 had extensive calcifications with deposits found throughout the brain, including the cerebellum, thalamus, midbrain and cerebral cortex (**Figures 9E–H**). Lastly, WT and GFAP-IL6 mice that were acutely treated with PLX5622 had comparable Iba1^+^ microglia numbers to untreated mice (**Figures 9C, D, I–K**). By contrast, there were fewer microglia in the brain of acute PLX5622-treated GFAP-IFN mice compared with untreated GFAP-IFN mice in the cerebellum, cortex and hippocampus. In the cortex and hippocampus of PLX5622-treated GFAP-IFN mice, the reduction of the number of microglia was statistically significant. Taken together, acutely treated GFAP-IL6 mice, in which the microglia compartment recovers from depletion, have a comparable molecular and clinical phenotype to untreated GFAP-IL6 mice. On the other hand, acute PLX5622 treatment largely recapitulates the molecular and clinical phenotypes of chronic PLX5622-treated GFAP-IFN mice.

## Discussion

We recently demonstrated that the microglia responses to IL-6 and IFN-α are stimulus-specific and give rise to cells with unique characteristics (44). Here we used the CSF1R inhibitor PLX5622 to determine whether the phenotypic differences of the microglia were indicative of distinct changes to their functional roles. While the cytokine environment driven by IL-6 signaling triggered rapid microglia repopulation following acute ablation, the cytokine environment driven by IFN-α did not allow microglia to repopulate. Importantly, our findings demonstrate that microglia protect the brain from excessive damage caused by chronic IL-6 or IFN-α-induced neuroinflammation.

### The ability of microglia to recover from acute ablation is divergently altered by IL-6- versus IFN-α-induced neuroinflammation

While microglia were acutely depleted throughout the brain following CSF1R inhibition in WT, GFAP-IL6 and GFAP-IFN mice, their ability to recover from ablation was dependent on the cytokine environment present in the brain. Surviving microglia in the brains of WT and GFAP-IL6 mice completely repopulated the brain. Interestingly, repopulation occurred more rapidly in GFAP-IL6 mice, suggesting increased presence of trophic factors in addition to IL-6, such as VEGFA, which is expressed more highly in GFAP-IL6 mice compared with WT and GFAP-IFN mice (44) and triggers microglia proliferation (73). By stark contrast, surviving microglia did not recover or repopulate the brain of GFAP-IFN mice. While there is competitive colonization of the CNS by surviving microglia and infiltrating macrophages following diphtheria toxin-induced acute microglial cell ablation, which induces cytokine storm (74, 75), microglia depletion in WT mice by CSF1R inhibitors is not accompanied by enhanced expression of inflammatory cytokines (20) or increased presence of infiltrating macrophages (20, 22). Accordingly, the vast majority of Iba1^+^ cells in the brain of repopulated WT and GFAP-IL6 mice were TMEM119^+^, indicating that these cells were likely derived from resident microglia, consistent with earlier reports (22). In addition, the ability of microglia to recover from acute ablation was not due to the gain or loss of the expression of the key microglia growth factors CSF1 or IL-34 in the brain. The ability of microglia to recover from acute ablation may instead be affected by their response to IL-6 versus IFN-α. IL-6 induces microglial cell proliferation *in vitro* and *in vivo* (44, 76–78) and likely contributes directly to the more rapid and extensive repopulation observed in GFAP-IL6 mice. On the other hand, IFN-α and other type I IFNs, in general, suppress cell proliferation (79) and may prevent microglia from repopulating the brain. In addition, it is possible that PLX5622-induced toxicity, in combination with IFN-α, prevented microglia in GFAP-IFN mice from repopulating the brain. Interestingly, we recently demonstrated that microglia proliferate and undergo apoptosis in the brain of GFAP-IFN mice and express genes associated with both cell cycle transition and apoptosis (44), indicating that the molecular signals which suppress microglia proliferation in PLX5622-treated GFAP-IFN mice are complex. Overall, although the detailed mechanisms remain uncharacterized, these findings suggest that the milieu induced by IL-6 versus IFN-α imparts microglia with unique abilities to functionally recover from acute ablation.

### Chronic CSF1R inhibition is deleterious in GFAP-IL6 and GFAP-IFN mice

While chronic CSF1R inhibition had no overt effect on WT mice, PLX5622 treatment was detrimental in GFAP-IL6 and GFAP-IFN mice. This was similarly seen in acutely PLX5622-treated GFAP-IFN mice, which were treated with PLX5622 for 14 days and then left without treatment for 10 weeks. Notably, the presence of large calcifications in the brain was the main pathological difference observed in both PLX5622-treated versus untreated GFAP-IL6 and GFAP-IFN mice. It remains to be seen if calcification contributes to or is a consequence of neural cell death in the mice. Since chronic PLX5622-treated GFAP-IL6 mice had severe cerebellar calcifications in the absence of seizures or premature death, it is unlikely that the calcifications alone contributed to the increased morbidity and mortality of acutely or chronically PLX5622-treated GFAP-IFN mice. Instead, the exaggerated clinical disease in the depleted GFAP-IFN mice is reminiscent of a recent study from Badimon and colleagues, in which PLX5622-treated, but not untreated, WT mice have severe seizures upon exposure to neurostimulants (80). This study further demonstrated that microglia restrain excessive neuronal excitation by metabolizing ATP and releasing adenosine, which quells excitatory glutamatergic signaling (80). Interestingly, chronic IFN-α signaling induces accumulation of glutamate in the CNS (81–84) and triggers neuronal dysregulation *via* glutamate excitotoxicity, impairment of neurotrophic signaling, dendritic shortening and reduced synaptic plasticity (85–87). There was evidence of enhanced neuronal dysregulation in PLX5622-treated GFAP-IFN mice, with more widespread loss of Purkinje cells and molecular layer interneurons in close proximity to calcifications compared with untreated GFAP-IFN mice. It is therefore conceivable that microglia loss, in combination with IFN-α-mediated neurotoxicity and neuronal dysregulation, increases the susceptibility of GFAP-IFN mice to seizures and death.

It was surprising to observe extensive cerebral calcification in the cerebellum of PLX5622-treated GFAP-IL6 mice, since calcifications are a cardinal histopathological feature of cerebral interferonopathies (68). However, severe calcification in PLX5622-treated GFAP-IL6 and GFAP-IFN mice was not accompanied by further increases in the expression of IFN-α, IFN-regulated genes, or other inflammatory cytokines associated with calcification, such as IFN-γ or IL-12 (70–72). These observations are consistent with the notion of a minimal role for IFN in the enhanced deposition of calcium in the brain of both PLX5622-treated GFAP-IL6 and GFAP-IFN mice and provide further evidence that calcifications can arise independently of interferonopathy. Cerebral calcification in the absence of interferonopathy have been described in humans, such as patients with mutations in the sodium transport gene SLC20A2 (88, 89) or the collagen gene COL4A1 (90, 91). In addition, elevated serum levels of IL-6 are associated with coronary artery calcification in patients with chronic kidney disease (92) and rheumatoid arthritis (93). Taken together, our findings implicate IL-6 as a novel mediator of cerebral calcification in the brain under certain pathologic conditions, however, the mechanism by which this cytokine drives calcification, as well as its contribution to cerebellar calcification in general, remains to be established.

### Microglia restrain calcification induced by IL-6- and IFN-α-induced neuroinflammation

Although severe calcification in PLX5622-treated GFAP-IL6 and GFAP-IFN mice was not accompanied by augmented expression of cytokines known to induce calcification, the development of cerebral calcification in these animals correlated with the absence of microglia. Accordingly, since the microglia in GFAP-IL6 mice acutely treated with PLX5622 rapidly recovered and repopulated the CNS, these mice did not exhibit cerebellar calcifications. Importantly, some WT mice treated long-term with PLX5622 did have small calcium deposits in the brain (**Figures 4D, G**), indicating that microglia likely suppress calcium mineralization. While the pathways in microglia that may regulate tissue calcification remain unclear and are the focus of ongoing studies, it is tempting to speculate that PLX5622-treated WT mice which exhibited small calcifications had been conditioned in particular ways which induced calcification, for example, through a trauma or immune activation. Given these observations, it is likely that the absence of microglia, in combination with the neuroinflammatory milieu induced by chronic IL-6 and IFN-α signaling, triggers unrestrained cerebral calcification in the brain of GFAP-IL6 and GFAP-IFN mice. These findings are similar to observations in platelet-derived growth factor B hypomorphic (*Pdgfb^ret/ret^*) mice, which have vessel-associated calcifications and express genes associated with IL-6 and IFN-α signaling (94). In these animals, microglia depletion *via* PLX5622 treatment or microglia dysfunction *via* TREM2 deficiency results in enhanced vessel-associated calcification in the thalamus and midbrain (94). Taken together, our findings point to a novel function of microglia in actively restraining calcification in the brain.

### The ability of microglia to restrain calcification is impaired in GFAP-IFN mice

Interestingly, the response of microglia to IL-6 versus IFN-α also alters their ability to restrain calcification. Although untreated mice had intact microglia compartments, untreated GFAP-IFN mice spontaneously develop cerebral calcifications (42, 45), while untreated GFAP-IL6 mice did not show any evidence of calcification. These findings indicate that while microglia in GFAP-IL6 mice can effectively regulate the perturbed calcium environment in the brain, GFAP-IFN mice likely exhibit spontaneous calcification as a consequence of IFN-α-mediated defects in cell calcium handling and metabolism in microglia and/or other cells. This may be the result of differential expression of calcification-restraining factors. For instance, compared with GFAP-IFN mice, cerebellar microglia from GFAP-IL6 mice express significantly higher levels of *Spp1* [(44) and quantified in **Supplementary Figure 3A**]. *Spp1* encodes for osteopontin, a secreted phosphoprotein which actively promotes regression of peripheral ectopic calcifications *in vivo* (95). Following excitotoxic insult, mice lacking *Spp1* exhibit progressive secondary neurodegeneration and microcalcification (96). In the CNS, *Spp1* is predominantly expressed by microglia (97). Microglia-secreted osteopontin following ischemic stroke binds and opsonizes precipitated calcium in cell debris, promoting its phagocytosis and removal by microglia (98–100). Therefore, the differential expression of *Spp1* by microglia in GFAP-IL6 versus GFAP-IFN mice may possibly contribute to ineffective restriction of calcium deposition by microglia in GFAP-IFN mice. On the other hand, *Trem2* is not differentially expressed by microglia from GFAP-IL6 and GFAP-IFN mice [(44) and quantified in **Supplementary Figure 3B**]. Therefore, *Trem2* is unlikely to contribute to the divergent abilities of microglia to restrain calcification in GFAP-IL6 versus GFAP-IFN mice. Future studies should endeavor to characterize the mechanisms by which microglia restrain calcification growth, as well as determine how the response to IFN-α impairs the ability of microglia and other CNS resident cells to handle and metabolize extracellular calcium in the brain.

## Conclusions

In summary, here we demonstrate that chronic IL-6 versus IFN-α production in the brain alters the ability of microglia to functionally recover from acute ablation, with enhanced microglia repopulation in GFAP-IL6 mice and suppressed microglia repopulation in GFAP-IFN mice. These functional changes are most likely the outcome of the distinct responses of microglia to IL-6 versus IFN-α in concert with other cytokines. Furthermore, rather than being protective, chronic CSF1R inhibition is deleterious in both GFAP-IL6 and GFAP-IFN mice. Microglia actively restrain calcification as a result of chronic IL-6 or IFN-α signaling. Furthermore, microglia protect the brain against IFN-α-mediated neurotoxicity and neuronal dysregulation. Taken together, our findings suggest that microglia protect against IL-6- and IFN-α-induced damage in the brain and warn against the use of CSF1R inhibitors in a pro-inflammatory environment where detrimental consequences may occur.

## Data availability statement

The original contributions presented in the study are included in the article/**Supplementary Material**, further inquiries can be directed to the corresponding author/s.

## Ethics statement

All animal experiments were performed in compliance with the NSW Animal Research Act and its associated regulations and the 2013 NHMRC Australian code for the care and use of animals for scientific purposes. Ethical approval for the use of all mice was obtained from the animal ethics committee of the University of Sydney (2018/1428).

## Author contributions

PW, IC, and MH conceived the study. PW, IC, and MH designed the experiments. PW performed the experiments. PW and BV analyzed experimental results. PW, IC, and MH performed histopathological analysis. PW, IC, and MH wrote the manuscript. All authors contributed to the review and editing of the manuscript. All authors contributed to the article and approved the submitted version.

## Funding

This study was funded in part by a grant from the National Health and Medical Research Council of Australia to MH (APP2001543). PW was the recipient of an Australian Government Research Training Program scholarship.

## Acknowledgments

The authors acknowledge the statistical assistance provided by the Sydney Informatics Hub, a Core Research Facility of the University of Sydney.

## Conflict of interest

Authors MH and IC received funding from Ionis Pharmaceuticals for experiments in an unrelated study using the GFAP-IFN mice.

The remaining authors declare that the research was conducted in the absence of any commercial or financial relationships that could be construed as a potential conflict of interest.

## Publisher’s note

All claims expressed in this article are solely those of the authors and do not necessarily represent those of their affiliated organizations, or those of the publisher, the editors and the reviewers. Any product that may be evaluated in this article, or claim that may be made by its manufacturer, is not guaranteed or endorsed by the publisher.

## Abbreviations

ARS, Alizarin red S; CALB, Calbindin; CNS, Central nervous system; CSF, Cerebrospinal fluid; CSF1, Colony-stimulating factor 1; CSF1R, Colony-stimulating factor 1 receptor; GCL, Granule cell layer; GFAP, Glial fibrillary acidic protein; H&E, Hematoxylin and eosin; IL, Interleukin; IFN, Interferon; LFB&CV, Luxol fast blue and cresyl violet; ML, Molecular layer; NF-200, Neurofilament-200; NMOSD, Neuromyelitis optica spectrum disorder; PCL, Purkinje cell layer; PV, Parvalbumin; WM, White matter; WT, Wildtype.
