# Supplementary material for: Microglia shield the murine brain from damage mediated by the cytokines IL-6 and IFN-α
Source: Front Immunol. 2022 Oct 28;13:1036799. doi: 10.3389/fimmu.2022.1036799 (PMC9650248; doi:10.3389/fimmu.2022.1036799)
Supplement: Supplementary file 1 [file DataSheet_1.pdf]

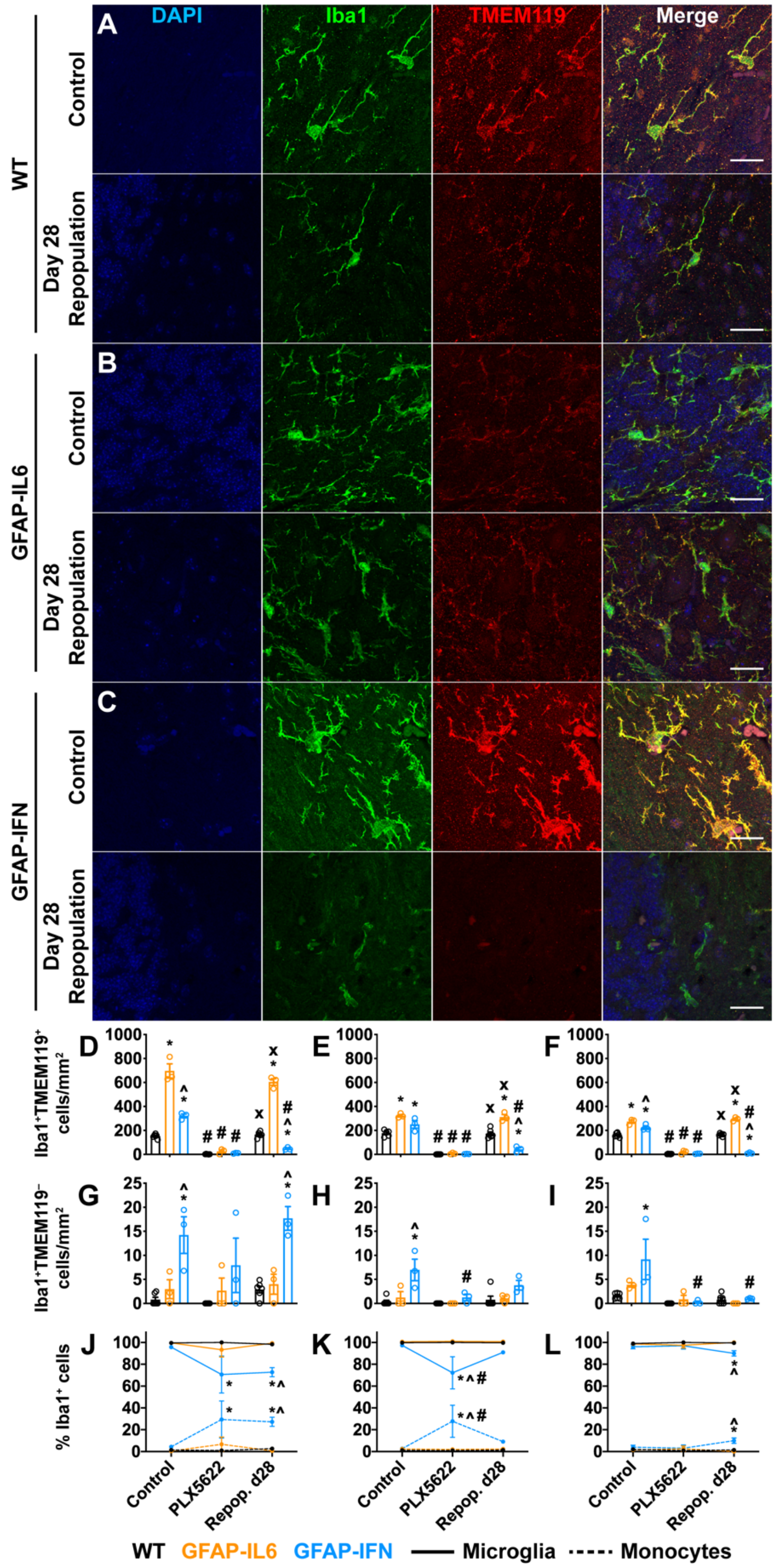

Supplementary Figure 1 Surviving microglia repopulate the depleted brain in WT and GFAP-IL6 mice but not in GFAP-IFN mice. **(A-C)** Representative immunofluorescence images (Iba1$^+$ microglia/macrophages, green; TMEM119$^+$ microglia, red; DAPI, blue) from the cerebellum of WT **(A)**, GFAP-IL6 **(B)** and GFAP-IFN **(C)** mice given control or PLX5622 diet for 14 days (repopulation day 0) or following 28 days after removal of PLX5622 (repopulation day 28). Scale bars, 20 µm. (**D-L)** Quantification of **(D-F)** the number of

Iba1^{+}TMEM119^{+} microglia per mm^2, **(G-I)** the number of Iba1^{+}TMEM119^{-} monocytes/macrophages per mm^2 and **(J-L)** the percentage of Iba1^{+}TMEM119^{+} microglia versus Iba1^{+}TMEM119^{-} monocytes/macrophages in the **(D, G, J)** cerebellum, **(E, H, K)** cortex and **(F, I, L)** hippocampus. In **(J-L)**, solid lines represent Iba1^{+}TMEM119^{+} microglia and dashed lines represent Iba1^{+}TMEM119^{-} monocytes/macrophages. n=3-6 mice/group. For **(D-I)**, graphs show individual values per mouse and mean ± SEM and for **(J-L)**, graphs show mean ± SEM. *, $p<0.05$ compared with WT of same diet; ^, $p<0.05$ compared with GFAP-IL6 of same diet; #, $p<0.05$ compared with untreated control of same genotype; x, $p<0.05$ compared with PLX5622 day 0 of same genotype using two-way ANOVA with Tukey's post-test.

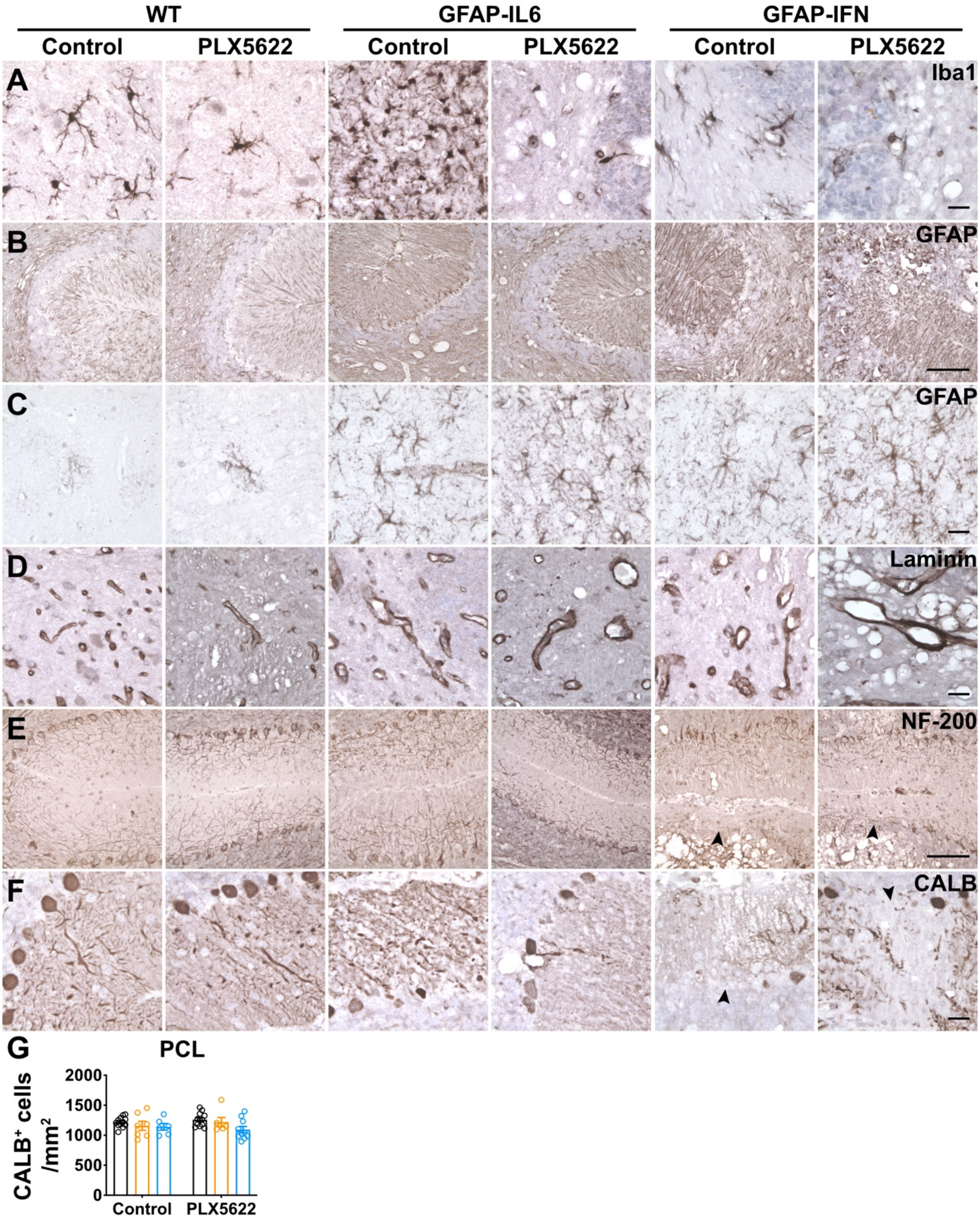

Supplementary Figure 2 Additional images of histopathology in the brain of GFAP-IL6 and GFAP-IFN mice following PLX5622 treatment. **(A-F)** Representative images of the **(A, B, D-F)** cerebellum and **(C)** cortex of mice treated with control or PLX5622 diet for 12 weeks. **(A)** Iba1, **(B, C)** GFAP in the **(B)** cerebellum and **(C)** cortex, **(D)** laminin, **(E)** neurofilament-200 (NF-200) and **(F)** calbindin (CALB) in the cerebellum. Black arrowheads indicate areas of neuronal cell loss. Scale bars for **(A, C, D, F)**, 20 µm. Scale bars for **(B, E)**, 100 µm. **(G)** Quantification of the number of $CALB^+$ Purkinje neurons per mm^2 in the Purkinje cell layer (PCL) of the cerebellum. Graph shows individual values per mouse and mean ± SEM. n=6-12 mice/group.

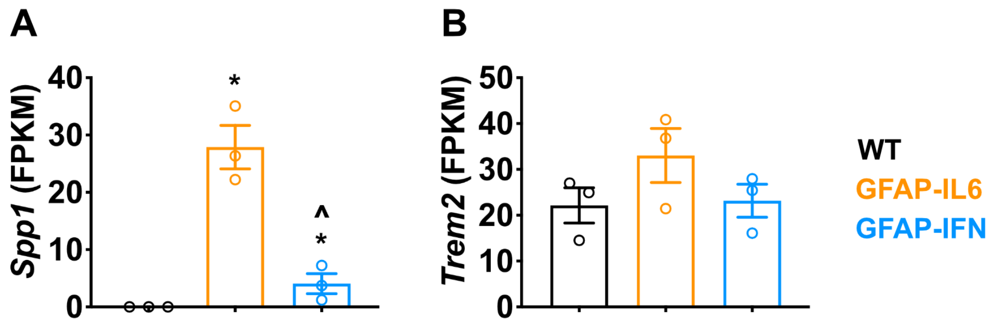

Supplementary Figure 3 Differential expression of *Spp1*, but not *Trem2*, by microglia in GFAP-IL6 versus GFAP-IFN mice. **(A-B)** Quantification of RNA-seq reads (fragments per kilobase of transcript per million mapped reads (FPKM)) for **(A)** *Spp1* and **(B)** *Trem2* in microglia isolated *ex vivo* from the cerebellum of 1-month-old WT, GFAP-IL6 and GFAP-IFN mice [1]. *, $p<0.05$ compared with WT; ^, $p<0.05$ compared with GFAP-IL6 using edgeR. n=3 mice/group.

References

1. West PK, McCorkindale AN, Guennewig B, Ashhurst TM, Viengkhou B, Hayashida E, et al. The cytokines interleukin-6 and interferon-α induce distinct microglia phenotypes. J Neuroinflammation. 2022;19(1):96.
